# Possible Function of Molecular Chaperones in Diseases Caused by Propagating Amyloid Aggregates

**DOI:** 10.3389/fnins.2017.00277

**Published:** 2017-05-16

**Authors:** Vladimir F. Lazarev, Elena R. Mikhaylova, Irina V. Guzhova, Boris A. Margulis

**Affiliations:** Laboratory of Cell Protection Mechanisms, Institute of Cytology of the Russian Academy of SciencesSt. Petersburg, Russia

**Keywords:** conformational neurodegenerative pathologies, cell-to-cell transmission, molecular chaperones, aggregation, prions

## Abstract

The vast majority of neurodegenerative pathologies stem from the formation of toxic oligomers and aggregates composed of wrongly folded proteins. These protein complexes can be released from pathogenic cells and enthralled by other cells, causing the formation of new aggregates in a prion-like manner. By this mechanism, migrating complexes can transmit a disorder to distant regions of the brain and promote gradually transmitting degenerative processes. Molecular chaperones can counteract the toxicity of misfolded proteins. In this review, we discuss recent data on the possible cytoprotective functions of chaperones in horizontally transmitting neurological disorders.

## Introduction

Conformational neurodegenerative pathologies, including Alzheimer's disease (AD), Parkinson's disease (PD), amyotrophic lateral sclerosis (ALS), and Huntington's disease (HD), arise from the formation of oligomers, or amyloidogenic aggregates of mutant proteins, either in or adjacent it neural cells. It is well established that mutant and damaged cellular polypeptides form first small oligomers with pronounced toxicity and then bigger polypeptide complexes with fibrillar or amorphous spatial structures. Data accumulated in recent years indicate that oligomeric and aggregated species may appear in the extracellular space, threatening the viability of nearby healthy cells (Lim and Yue, 2015). This phenomenon is similar to prion propagation process, where a protein with an incorrect conformation is transmitted from a sick cell to a healthy one using various transport mechanisms. In the last decade, multiple protein pathogens (including huntingtin, alpha-synuclein, tau, and SOD1) have been shown to have prion-like properties (Holmes and Diamond, 2012). In this review, we focus particularly on the propagation of non-classic prions, rather than of typical yeast prions, that constitute two separate species which besides a few similarities exhibit distinctions in mechanisms of their assembly, transportation and particularly conversion of target proteins to aggregation-prone form.

**Figure 1:**
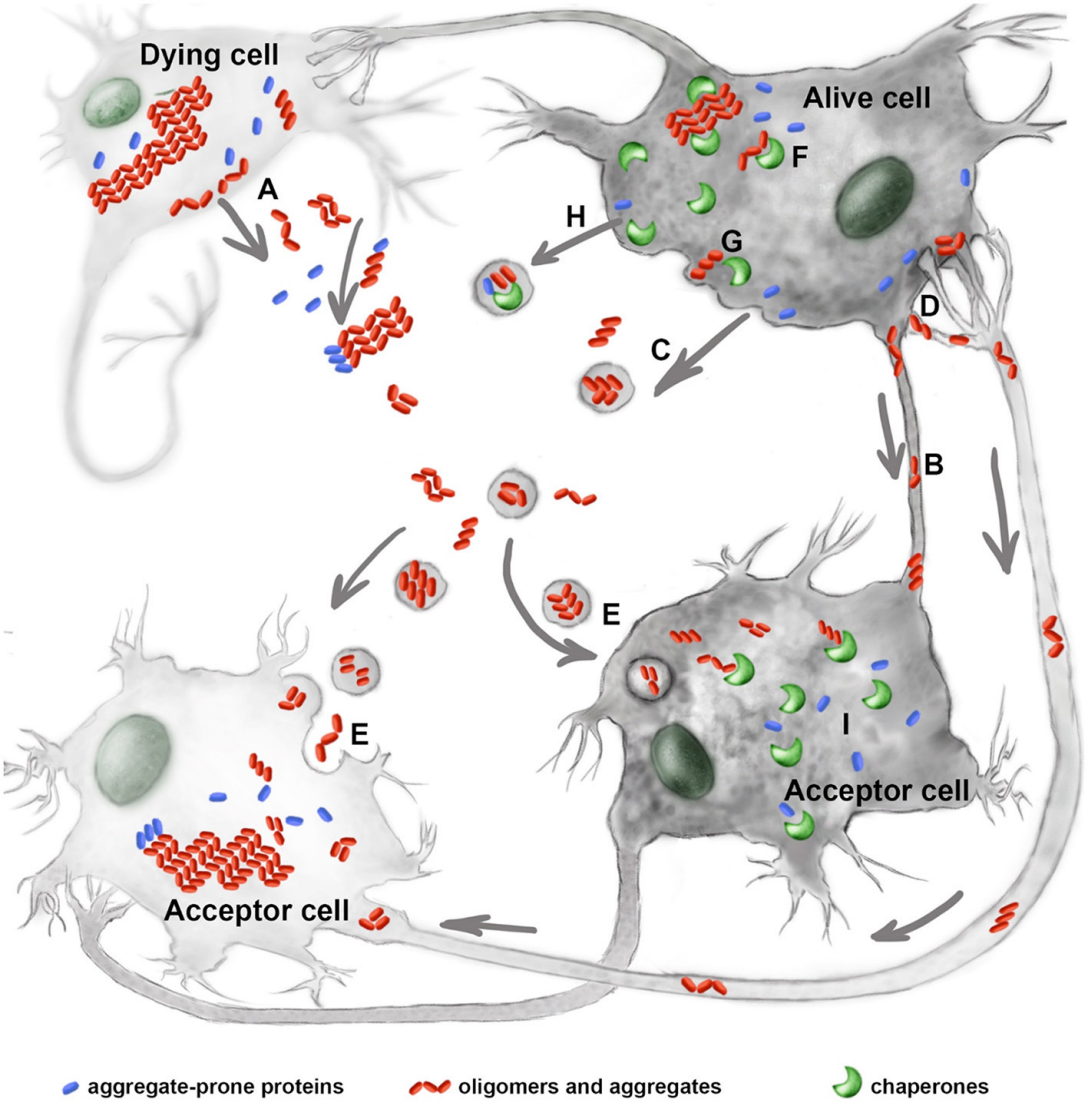
**Molecular chaperones interfere with the prion-like process of neurological disorders**. Mutant proteins, their oligomers and aggregates leave the damaged **(A)** or alive cells using tunneling nanotubes **(B)**, exosomes **(C)**, or trans-synaptic contacts **(D)**. The extracellular protein complexes penetrate inside acceptor cells by employing endocytosis **(E)**. Molecular chaperones, Hsp70, Hsp40, Hsp110, and other restrict aggregate growth in donor cells **(F)** and assist in the release of pathogenic proteins from the latter **(G)**; chaperones of the Hsp70 family were also found to accompany mutant proteins in exosomes **(H)**. In acceptor cells the chaperones play dual role of modulating prion-like process of aggregation **(I)**. See text for details.

Horizontal transmission of pathology is a complicated, multiphasic process that lasts years or decades. The first stage of this process is the accumulation of oligomers, aggregates, or both. Although, some of the cells producing misfolded proteins die, others are able to survive because of properly functioning autophagy, proteasomes, or chaperones (Brandvold and Morimoto, 2015; Figure 1). In the second disease stage, oligomeric (and larger) complexes are released from damaged or living neurons. Such structures are found in the cerebrospinal fluid, plasma, saliva, and urine and can be used as disease markers.

The final stage of pathology propagation involves the interaction of migrating complexes with an acceptor cell. The pathogenic proteins can penetrate healthy cells and initiate the formation of secondary aggregates in new hosts. This phenomenon has been observed in cells incubated with tau, α-synuclein, or polyglutamine-containing proteins (Holmes and Diamond, 2012; Figure 1).

Molecular chaperones, often mistakenly named as heat shock proteins (Hsps), have been shown to protect neural cells from numerous pathogenic factors, including those causing neurodegeneration; this is convincingly proved by data obtained from hundreds of cell and animal models. In earlier studies, these protective effects were accounted for by chaperones functioning as anti-aggregation machinery within cells. However, more recent studies have shown that some chaperones, particularly relating to Hsp70 family (HSPA1A and HSPA8), can participate at other stages of the prion-like process of disease transmission. Below we review recent data on the mechanisms of intercellular propagation of neurological pathologies and discuss the possible involvement of chaperones. We consecutively discuss the formation of aggregating protein species within cells, then their persistence in the extracellular matrix, and finally the penetration of target cells (Figure 1).

## Protein pathogens meet chaperones in a neural cell

Irrespective of their origin, mutant or damaged proteins tend to form oligomers and amyloid-like aggregates inside cells. These aggregates are insoluble in high-molar salt solutions or even in sodium dodecylsulfate, as was observed for the PrPsc prion and mutant huntingtin proteins (Leffers et al., 2005; Natalello et al., 2011). The exclusively high stability of pathogenic complexes is usually explained by their structure: according to a well-established theory, the aggregates consist of beta-sheets forming dense stacks by H-bonds (Perutz et al., 2002). An alternative theory is that such stability arises from the formation of covalent bonds between pathogenic proteins and other cellular polypeptides; such complexes are formed between polyglutamine long chain-containing proteins and glyceraldehyde-3-phosphatedehydrogenase (GAPDH) (Orru et al., 2002; Guzhova et al., 2011).

It is generally accepted, but not well established by experimental data that monomers and oligomers of mutant or damaged proteins are toxic because they cause damage to multiple ion channels and inactivate polypeptides that participate in all basic cellular functions, transport, enzymatic reactions, and transcription (Margulis et al., 2013; Verma et al., 2015). In contrary, the already formed aggregates are thought to be less harmful to cells probably because they possess smaller active surface area to interact with and to harm other cellular proteins than their low molecular weight homologs (Kim et al., 2016; Figure 1). Additionally, the careful analysis of data obtained from cells overproducing mutant superoxide dismutase or huntingtin suggests that the aggregates of these polypeptides threaten intracellular transport mechanisms and almost completely inhibit proteasomal and lysosomal machinery (Rubinsztein, 2006; Seiliez et al., 2014).

Among the multiple mechanisms that a cell can use to cope with growing aggregates, chaperones appear most efficient (Lindberg et al., 2015). Chaperones are able to recognize and bind misfolded or damaged polypeptides and recover them through sequential binding-release cycles in ATP-dependent manner. In these activities proteins of Hsp70 family (HSPA group, according to classification of Kampinga et al., 2009) including Hsp70 inducible (HSPA1A) and Hsc70 constitutively synthesized (HSPA8) forms play a pivotal role in physiology of stressed and normal cells, respectively. So-called co-chaperones, such as members of the DNAJ family and nucleotide-exchanging factors belonging to the Bag or Hsp110 protein families are also involved in ATP-dependent chaperone cycles (Zuiderweg et al., 2017). Hsp70 with its DnaJ-like aide have been shown to inhibit the growth of aggregates of huntingtin and the other proteins with long polyglutamine repeats (Labbadia et al., 2012), α-synuclein in a model of PD (Auluck et al., 2002), tau in AD (Zhang et al., 2008), and two key pathogens in ALS, SOD1, and TDP-43 (Koyama et al., 2006; Udan-Johns et al., 2014). Interestingly, the Hsc70 cognate chaperone, one of the J-proteins, DNAJB1, and the Hsp110 family nucleotide exchange factor form an ATP-dependent machine that disassembles α-synuclein amyloids, converting them into non-toxic monomers (Gao et al., 2015). The same group has shown also that for the efficient dissociation of amyloid aggregates synergic cooperation of DNAJA and DNAJB is necessary in both nematode and human cells (Nillegoda et al., 2015). In a majority of cell and animal models, anti-aggregation activity based on the Hsp70 chaperone resulted in numerous cytoprotective effects, correction of behavioral abnormalities, amelioration of motility function, and an increase in life expectancy (Leak, 2014; Pratt et al., 2015).

Small Hsps are another family of chaperones involved in the process of proteotoxic pathology. Small Hsps have been shown to efficiently counteract the aggregation of many pathogenic proteins, including α-synuclein, huntingtin, and ataxins. The importance of these chaperones was demonstrated in studies showing that the hereditary mutations in HSPB1/Hsp27 and the associated reduced ability to maintain proteostasis gave rise to a great number of neurodegenerative disorders, including Charcot-Marie-Tooth disease and ALS (Echaniz-Laguna et al., 2017).

Generally, in the majority of proteotoxic diseases, cellular chaperones were shown to protect neurons from the action of mutant protein oligomers and aggregating species; the number of such reports is in the hundreds (Yerbury et al., 2016). Reduced cellular chaperone content of function causes disease progression (Ogawa et al., 2013). A weakening of chaperone expression has also been reported; in HD and AD patient brain tissues, the expression of approximately 30% of more than 300 chaperones was reduced, which matched well with the data obtained for aged persons (Brehme et al., 2014). Since most proteotoxic diseases are typically associated with age, the decline of heat shock response, chaperone efficacy, and other mechanisms of protein quality control might complicate efforts to correct these disorders with the aid of chaperones (Ben-Zvi et al., 2009; Labbadia and Morimoto, 2015).

## Migrating pathogenic proteins and extracellular chaperones

Cells producing mutant proteins in the form of cytotoxic oligomers or large aggregates can survive if they have well-developed systems of protein quality control, or they might die. In the latter case, all cellular proteins including pathogenic polypeptides would be released and occur in the extracellular space for an undefined period. Although, the damage to the plasma membrane and leakage of the cytosolic proteins appears to be the most probable route for the/accumulation of pathogenic protein complexes, this has been demonstrated only for long polyglutamine repeats (Ren et al., 2009; Mikhaylova et al., 2016; Figure 1). Viable cells have also been shown to excrete polyglutamine long repeat polypeptide, and Hsp40 (DNAJ class protein) over-expression in mouse neuroblastoma cells inhibits extracellular transport of the pathogenic protein (Popiel et al., 2012). Another protein pathogen, TDP-43, was found to be transported from viable cells with the assistance of Hsc70 (HSPA8), and its J-domain co-chaperone DnaJC5; the data favored the assumption that the extracellular transport of TDP-43, tau, and α-synuclein is performed via a SNAP-23-dependent exocytosis mechanism, relative to the ubiquitous SNARE transporting complex (Fontaine et al., 2016).

Many protein pathogens employ exosomes as the universal mechanism of intercellular transport, and such particles carrying beta-amyloid and α-synuclein have been found in the conditioned medium of cell culture models of AD or PD, as well as in samples of body fluids of neurological patients (Soria et al., 2017). Recently, Hsp70 and Hsp40 were also identified in exosomes, and the addition of these chaperone-rich particles to cells expressing proteins with long polyglutamine chains resulted in the suppression of aggregation of the pathogen. These data show that chaperones can inhibit the toxicity of protein pathogens inside a neural cell and outside it (Takeuchi et al., 2015).

Direct synaptic contacts are another mechanism of intercellular transmission of pathology; these routes are employed by beta-amyloid and tau, as demonstrated by Meyer-Luehmann et al. (2006) and Mohamed et al. (2013) respectively. Additionally, “classic” prions were also shown to use tunneling nanotubes for their intercellular transport, thus expanding the variety of mechanisms employed by propagating pathogenic proteins (Gousset et al., 2009). The role of molecular chaperones in the latter types of transmission is elusive.

The application of high-resolution methods to the analysis of extracellular pathogenic proteins led to the conclusion that, in all body fluids studied to date, the content of at least four protein pathogens (beta-amyloid, α-synuclein, tau, and TDP-43) was directly proportional to the age of a patient or severity of the disease, thus confirming that these exogenously transmitted polypeptides threaten those cells that are adjacent to the pathological cells (Verstraete et al., 2012; Cohen et al., 2016; Ruan et al., 2016; Shahnawaz et al., 2017). Although, most of the results demonstrating the danger of migrating misfolded proteins were obtained using oligomers or fibrils made of purified polypeptides, it should be emphasized that, during transmission from one cell to another, these polypeptides can sequester other molecules from their parent cell. Recently, we demonstrated that mutant huntingtin released from dying cells is accompanied by GAPDH, which increases the toxicity and cell-penetrating ability of polyglutamine (Mikhaylova et al., 2016). Migrating complexes may contain molecular chaperones, and this has been demonstrated in experiments with α-synuclein, which, as shown in a series of elegant experiments, tends to form oligomers both inside and outside neural cells (Danzer et al., 2011). The authors found that Hsp70, using its chaperone activity, reduced the formation of an extracellular oligomer of α-synuclein and its toxicity. Overall, the data presented in this section show that molecular chaperones function in neural cells affected by a protein pathogen, as well as in the extracellular space.

## Role of chaperones at the final stage of prionization

The final stage of prion-like pathology transmission includes the reaction of a protein pathogen with a target cell, its permeabilization, and the formation of the aggregates in a new host (Figure 1). The separate events occurring in this pathogenic chain are well-documented, and the many mechanisms for the intracellular transport of protein pathogens have been described in both cellular and animal models (Holmes and Diamond, 2012). The diverse character of the transport mechanisms may be illustrated by the fact that α-synuclein and mutant SOD1 can use either endocytosis or macropinocytosis on route to their target cell (Lee et al., 2011; Munch et al., 2011). The role of chaperones during this penetration stage remains unclear, though Hsp70 itself appears to be an example of a protein that can easily cross the plasma membrane in both directions; to do that Hsp70 has been shown to employ multiple mechanisms, including formation of individual pores in the bilayer membrane (De Maio, 2014) and classic receptor-mediated endocytosis (Shevtsov et al., 2014). The high cargo-transporting capacity of Hsp70 was demonstrated in our study, where we have shown that a 20-mer peptide derived from the chaperone sequence carrying avidin and anti-Hsp70 antibody was able to penetrate inside living cells and reduce their anti-apoptotic resistance by neutralizing self Hsp70 (Komarova et al., 2015). Interestingly, Couceiro with coworkers discovered the sequence-specific and size-dependent internalization of aggregating peptides; the internalization requires the tuning of cellular proteostasis mechanisms, including additional expression of Hsp70 (HSPA1A) through the activation of Hsf-1. The authors suggest that the differences in cellular response contribute to the particular role of specifically aggregated proteins in disease (Couceiro et al., 2015).

The terminal point of an intercellular propagation of proteotoxic pathology is the prionization of normal cellular polypeptides and correspondingly infection of formerly healthy cells (Figure 1). The prionization mechanism, the transfer of a misfolded structure to a subsequent polypeptide molecule, has been thoroughly described for the best-known prions (e.g., PrP), and it has been concluded that, despite some similarities between classic prions and those causing tauopathies, synucleopathies, and others, there are many distinctions (Vázquez-Fernández et al., 2017). The function of chaperones during the prionization of tau, α-synuclein, SOD1, and other protein pathogens remains disputable. As discussed above, the Hsc70 (HSPA8), together with J-domain-containing polypeptides, might help oligomers or aggregates of misfolded proteins to release these from living cells (Fontaine et al., 2016) and can deliver the polyglutamine-rich polypeptide inside a cell (Takeuchi et al., 2015). Once entering an acceptor cell, pathogenic polypeptides recruit intracellular proteins, homologic, or non-homologic, to prionize them. Prionization effect has been demonstrated for tau, α-synuclein, SOD1, TDP-43, and polyglutamine, however, whether this mechanism involves the mobilizing of monomers of misfolded proteins and through the formation of various forms of aggregates (as happens in case of “real” prions) needs further investigation (Stroylova et al., 2012; Bose and Cho, 2017). Since molecular chaperones have been convincingly shown to defend those cells in which the pathogenic oligomers and aggregates are initially formed, as well as cells secondarily infected by prion-like pathogens, we suggest that the activation of molecular chaperones is a potent instrument for coping with transmitting proteotoxic pathologies.

## Activating chaperones in prion-like pathologies

The activation of heat shock response is currently employed in a variety of pathogenic simulations, including heart injury and cancer therapy. In this respect, moderate to high frequency of sauna bathing (in other words whole-body heat shock) does not seem to be an exotic sort of prophylaxis since it was associated with lowered risks of dementia and Alzheimer's disease (Laukkanen et al., 2017). To date, the most appropriate and safe methods in the Hsps-mediated therapy are pre-conditioning, localized hyperthermia, hyperbaric treatments, and natural substances known to induce Hsps and to maintain a normal level of proteostasis (e.g., resveratrol) (Putics et al., 2008). Among the well-known inducers of Hsps are inhibitors of Hsp90, geldanamycin, and compounds that, by binding ATP-ase domain of the chaperone, reduce its ability to stabilize multiple client proteins and lead to the inhibition of pro-cancer signaling systems. A few Hsp90 inhibitors were found to activate heat shock response through the up-regulation of Hsf1; this transcription factor serves as a client protein of the chaperone, and one of the most strongly induced proteins is Hsp70 (Bose and Cho, 2017). Because of their Hsp70-inducing activity, many inhibitors of Hsp90 have been shown to ameliorate pathological manifestations in several models of neurodegeneration. For example, 17-(allylamino)-17-demethoxygeldanamycin (17-AAG, tanespimycin) is effective against various polyglutamine diseases, both in transgenic flies and mice (Table 1). 17-AAG was also found to reduce α-synuclein oligomerization and its toxicity in a cultured cells model of PD. Another geldanamycin analog, 17-dimethylaminoethylamino-17-demethoxy-geldanamycin (17-DMAG, alvespimycin), is more soluble in water solutions and can pass the blood-brain-barrier and, therefore, its application in therapeutic schedules looks more promising. 17-DMAG administration in transgenic mice with spino-bulbular muscular atrophy (SBMA) reduced the content of the most cytotoxic monomeric and oligomeric species of androgen receptor and ameliorated motor dysfunction in the animals (Tokui et al., 2008). The augmentation of motoric function was also reported when 17-DMAG was applied to other polyglutamine disease models, Machado-Joseph disease or spino-cerebellar ataxia type 3 (Silva-Fernandes et al., 2014; Table 1). Celastrol, quinone methide triterpene was also demonstrated to inhibit Hsp90 activity by binding to the C-terminal domain of the chaperone; this compound protects the cells from protein aggregation and cytotoxicity in models of ALS, HD, and PD (Table 1). The compound SNX-2112 was found to bind to the ATP-binding pocket of Hsp90 and induce Hsp70 synthesis in brain cells. In experiments with cells simulating PD, treatment with SNX and its derivatives was found to decrease α-synuclein oligomerization and toxicity (McFarland et al., 2014).

**Table 1:** **Chaperone drugs to cure proteotoxic diseases**.

**Name of a substance**	**Principle of action**	**Pathology/effect**	**References**
17-AAG	Hsp90 inhibitor/activation of HSR	Spinocerebellar ataxia/Transgenic flies PD/cell model	Fujikake et al., 2008; Danzer et al., 2011
17-DMAG	Hsp90 inhibitor/activation of HSR	Machado-Joseph disease/Transgenic CMVMJD135 mice	Silva-Fernandes et al., 2014
SNX-related compounds	Hsp90 inhibitor/activation of HSR	PD/Rat model	McFarland et al., 2014
Celastrol	Hsf1 activation	ALS/Transgenic mice Dentatorubral pallidoluysian atrophy/cell model PD/Transgenic flies	Kiaei et al., 2006; Zhang and Sarge, 2007; Faust et al., 2009
Arimoclomol	Co-inducer of HSR	ALS transgenic mode	Kalmar et al., 2008
Geranylgeranylacetone	Hsf1 activation	SMBA/Transgenic flies AD/Transgenic APP23 mice	Katsuno et al., 2005; Hoshino et al., 2013
YM-08	Hsp70 inhibitor	Taupathy (AD)/Brain slices	Miyata et al., 2013
Human Hsp70	Chaperonic activity	ALS/Transgenic mice AD/Transgenic 5xFAD mice	Gifondorwa et al., 2007; Bobkova et al., 2014

The balance between useful and detrimental effects of the chaperone inhibitors is subtle, since suppressing one chaperone system, for instance based on Hsp90, one may affect another subcellular structure and cause side effects, cytotoxicity, that sometimes was observed when high doses of geldanamycin were applied.

The list of powerful Hsf1 activators includes geranylgeranylacetone, which inhibited the nuclear accumulation of pathogenic polyglutamine-containing androgen receptors in a mouse SBMA model and decreased the amount of beta-amyloid plaques and synaptic loss in an AD model (Table 1). A few low molecular weight inhibitors of Hsp70 have also been shown to ameliorate the attributes of neurodegeneration associated with the proteotoxicity of misfolded proteins. One such substance, YM-8, was shown to bind to Hsp70, and reduce phosphorylated tau levels in cultured brain slices; the mechanism of such action remains unclear (Miyata et al., 2013).

In addition to small molecules, the chaperones themselves have gained much attention. Intraperitoneally injected recombinant Hsp70 was shown to increase lifespan, delay symptom onset, and preserve motor function in G93A mutant SOD1 mice (Table 1). Similar neuroprotective effects have been shown for pure Hsp70 applied in a 5XFAD transgenic mouse model of AD; the intranasal delivery of the chaperone was found to reduce beta-amyloid plaque accumulation and to diminish losses of spatial memory in animals (Table 1).

Here, we have mainly discussed drug-like compounds shown to function in those cells that are donors of protein pathogens. However, it is our hope that such compounds might also exert protective effects at other points of the pathogenesis.

## Conclusion

During the last decade, our knowledge of neurologic diseases has been enriched by the demonstration of the prion-like character of tens of pathologies, starting from the release from initially pathologic cells to infection of adjacent cells. Since molecular chaperones play an important role in these events, novel drugs that target Hsps in the assembly of extracellular particles and their extra- and intracellular transport will be necessary.

## Author contributions

VL, BM, and IG wrote the review, EM presented the Figure 1 and the Table 1.

## Funding

This work was supported partly by RFBR grant 16-38-60196 mol_a_dk (VL), Award of the President of the Russian Federation MK-8274.2016.4 (EM) and by the Grant of Russian Science Foundation (RSCF) No 14-50-00068 (IG, BM).

### Conflict of interest statement

The authors declare that the research was conducted in the absence of any commercial or financial relationships that could be construed as a potential conflict of interest.
